# Structure-Based Simulations for Folding of a tRNA Isodecoder

**DOI:** 10.3390/molecules31101555

**Published:** 2026-05-07

**Authors:** Lev Levintov, Esteban A. Orellana, Harish Vashisth

**Affiliations:** 1 Department of Chemical Engineering and Bioengineering, University of New Hampshire, Durham, NH 03824, USA; lev.levintov@unh.edu; 2 Department of Molecular and Systems Biology, Geisel School of Medicine at Dartmouth College, Hanover, NH 03755, USA; esteban.orellana@dartmouth.edu; 3 Dartmouth Cancer Center, Dartmouth College, Lebanon, NH 03756, USA; 4 Department of Chemistry, University of New Hampshire, Durham, NH 03824, USA; 5 Integrated Applied Mathematics Program, University of New Hampshire, Durham, NH 03824, USA; 6 Molecular and Cellular Biotechnology Program, University of New Hampshire, Durham, NH 03824, USA

**Keywords:** isodecoder, MD simulations, N^2^,N^2^-dimethylguanosine modification, tRNA

## Abstract

The $N^{2}$,$N^{2}$-dimethylguanosine ($m^{2,2}G$) modification at the G27 nucleotide of transfer RNA (tRNA) is a crucial modification known to impact tRNA folding. Although some functional implications of $m^{2,2}G$ have been characterized, little is known about the molecular details of the effect of this modification on tRNA folding. In this work, we study folding of a tRNA isodecoder molecule by conducting all-atom structure-based simulations initiated from an ensemble of unfolded configurations. We observed that the folding of the modified tRNA proceeds cooperatively and hierarchically, beginning with the folding of the anticodon stem loop, followed by the folding of the D-stem loop or T-stem loop, and ending with the folding of the acceptor stem, thereby resulting in a fully folded configuration. However, the folding of the unmodified tRNA isodecoder revealed partially-folded intermediate configurations to be more favorable than a fully folded state. These results provide insights into the role of methylation in the folding of the tRNA isodecoder studied here and have broader implications for the folding of other tRNA species.

## 1. Introduction

Transfer RNA (tRNA) is a short non-coding RNA responsible for decoding codon triplets in messenger RNA (mRNA), delivering the correct amino acids to the ribosome, thus playing a central role in protein synthesis [1,2,3]. Thus, any dysregulation of tRNA function through mutations or overexpression may lead to various diseases [4,5] because the structure and folding properties of tRNA are highly regulated in cells [5,6,7]. Given the central role of tRNA in diverse biological processes, it has also been recently proposed as a promising therapeutic target to modulate protein synthesis, thereby enabling novel opportunities to intervene in human health [5,8,9].

The tRNA epitranscriptome [10] has been identified as a key regulatory mechanism with numerous post-transcriptional modifications that impact the structure, stability and folding of tRNA [5,6,7,11,12,13,14]. Among these, the $N^{2}$,$N^{2}$-dimethylguanosine ($m^{2,2}G$) modification is one of the most commonly observed modifications in all tRNA species, thus highlighting its functional relevance in tRNA [15,16]. Specifically, the $m^{2,2}G$ modification is deposited on the G26 or G27 nucleotide (or both) and has been suggested to affect the folding of tRNA, with significant implications for protein synthesis [16,17,18,19].

**Figure 1:**
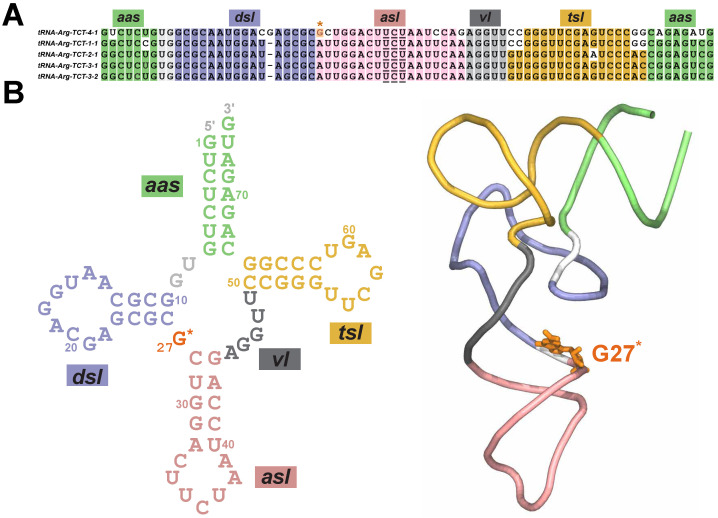
**Sequence and structure of ${\mathrm{tRNA}}^{\mathrm{Arg}}$**. (**A**) The sequence alignment of the mature tRNA-Arg-TCT isodecoders with conserved residues and conserved anticodon nucleotide triplet (UCU; underlined) highlighted in unique colors based on the structural domain: amino acid acceptor stem (green; *aas*); anticodon stem loop (pink; *asl*); D-stem loop (blue; *dsl*); T-stem loop (yellow; *tsl*); and variable loop (gray; *vl*). The conserved methylated G27^*^ nucleotide in ${\mathrm{tRNA}}^{\mathrm{Arg}}$ (labeled as tRNA-Arg-TCT-4-1) isodecoder is highlighted in orange color and marked by the * symbol. (**B**) The secondary structure and the tertiary structure of ${\mathrm{tRNA}}^{\mathrm{Arg}}$ are shown with key structural domains uniquely colored and labeled as in (**A**).

A representative example of tRNA that highlights the functional role of chemical modifications is an arginine tRNA family (tRNA-Arg-TCT) [20]. This family consists of five mature isodecoders that share the same anticodon triplet (UCU) but differ in their sequences (Figure 1A) [14]. Such sequence diversity among isodecoders has been proposed to expand the functional capacity of the tRNA pool by enabling cells to fine-tune translation and adapt to environmental changes [5,14]. However, despite a high sequence similarity in the tRNA-Arg-TCT family, only tRNA-Arg-TCT-4-1 (hereafter termed ${\mathrm{tRNA}}^{\mathrm{Arg}}$) contains the G27 nucleotide having the $m^{2,2}G$ modification (Figure 1A) [7]. Furthermore, this isodecoder is uniquely expressed in the central nervous system (CNS) [5,21], where it has been proposed to regulate synaptic transmission [22]. Thus, its dysregulation or any mutation in the $m^{2,2}G27$ nucleotide (hereafter termed G27^*^; Figure 1B) has been linked to neurological disease and malignant transformation, further highlighting the importance of understanding how this modification influences the folding and function of tRNA [21,23]. Additionally, other modifications in tRNAs have been proposed to be linked to the region-specific development of neurological and oncogenic diseases, including various anticodon modifications (e.g., inosine, yW, $t^{6}$A, ${\mathrm{mcm}}^{5}$$s^{2}$U), $m^{7}$G, $m^{5}$C, etc. [5,24,25].

Although the role of modified G27 in the correct folding of tRNA has been explored through functional and aminoacylation studies [14,26,27], the molecular details of the mechanism of folding of ${\mathrm{tRNA}}^{\mathrm{Arg}}$ in the presence of this chemical modification remain unknown [5]. Previous molecular dynamics (MD) simulation studies of tRNA molecules have focused primarily on understanding how chemical modifications influence the dynamics and conformational flexibility of the folded tRNA structure [28,29,30,31,32,33,34,35,36]. These studies have suggested that a chemical modification can alter local interactions in which the modified base is directly involved, leading to a destabilized local base-pairing interaction or the formation of new interactions that improve the global stability of tRNA. However, no studies have reported the structural effects of modifications on tRNA folding and no simulation studies exist on tRNA isodecoders.

Although all-atom MD simulations can, in principle, be used to probe tRNA folding, applying this approach directly to tRNA remains computationally challenging. Folding of tRNA molecules generally occurs on timescales longer than milliseconds [26], requiring extremely long MD simulations on the millisecond timescale for spontaneous observation of a folding event, which is beyond the timescales routinely accessible to atomistic MD simulations, even with modern supercomputing hardware [37,38]. In comparison, native structure-based simulation techniques that utilize structure-based potentials have been successfully used to characterize the folding of various biomolecules [38,39]. These structure-based techniques include G$\bar{o}$-model simulations [40,41,42,43], in which the folding process is described by a funneled energy landscape with an ensemble of unfolded conformations progressively converging toward the native (folded) state. This approach has been widely validated and successfully applied to characterize the folding of proteins [44,45,46,47] and RNA molecules [48,49], effectively capturing different folding pathways, intermediate states, and transition states.

In this work, we study the impact of the $m^{2,2}G$ modification (G27G27^*^) on the folding of ${\mathrm{tRNA}}^{\mathrm{Arg}}$. Specifically, we conducted all-atom structure-based G$\bar{o}$-model simulations [40,41,42,43] to establish the folding mechanism of ${\mathrm{tRNA}}^{\mathrm{Arg}}$ with and without $m^{2,2}G$ modification. Using structure-based simulations is a promising approach for probing the folding mechanisms of tRNA molecules because it employs a well-defined reaction coordinate based on the native contacts while also retaining atomic resolution. We observed that the folding of the modified ${\mathrm{tRNA}}^{\mathrm{Arg}}$ proceeded via a cooperative and hierarchical mechanism toward a thermodynamically favored folded configuration. However, in the absence of the $m^{2,2}G$ modification, we observed that the intermediate partially-folded configurations of ${\mathrm{tRNA}}^{\mathrm{Arg}}$ were thermodynamically more favorable than both the unfolded and folded states.

## 2. Results

### 2.1. Folding of Modified ${\mathrm{tRNA}}^{Arg}$

${\mathrm{tRNA}}^{\mathrm{Arg}}$ adopts a characteristic L-shaped tertiary configuration comprised of two orthogonal helical segments that further consist of individual secondary structure domains (Figure 1B) [1,2]. Specifically, the amino acid acceptor stem (*aas*) domain is structurally located near the T-stem loop (*tsl*) domain, forming the acceptor region that is linked to the anticodon stem loop (*asl*) domain through the D-stem loop (*dsl*) and variable loop (*vl*) domains that define the “elbow” region (Figure 1B) [1]. The modified nucleotide G27, which is known to impact the folding of tRNA molecules [5,16], is located between the *dsl* and *asl* domains (Figure 1).

Using all-atom structure-based G$\bar{o}$-model simulations, we probed the effect of the modified nucleotide on the folding of ${\mathrm{tRNA}}^{\mathrm{Arg}}$. Specifically, we conducted 120 G$\bar{o}$-model simulations of ${\mathrm{tRNA}}^{\mathrm{Arg}}$, 60 simulations in each of the two states (modified and unmodified) starting with an unfolded configuration and terminating in a folded configuration. We defined the initial state for each structure-based simulation as a unfolded ${\mathrm{tRNA}}^{\mathrm{Arg}}$ configuration with no native contacts; 60 distinct unfolded configurations were used for each ${\mathrm{tRNA}}^{\mathrm{Arg}}$ state. The folded tRNA structure (Figure 1B) was used to define the native state.

**Figure 2:**
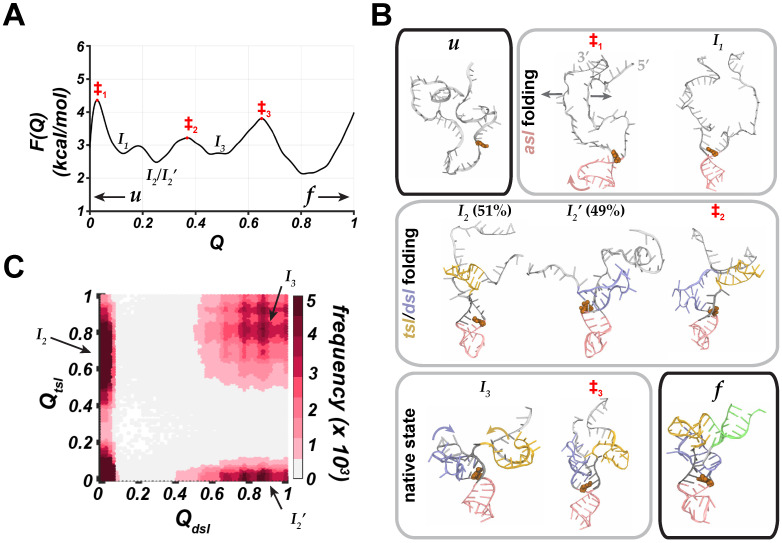
**Thermodynamics and conformational metrics for the folding of modified ${\mathrm{tRNA}}^{\mathrm{Arg}}$**. (**A**) Free energy profile as a function of the fraction of native contacts (*Q*) highlighting the transition from the unfolded state (***u***) to the folded state (***f***) via a series of intermediate (***I***) and transition (‡) states. (**B**) Snapshots from a representative structure-based simulation showing conformational rearrangements during the folding of the modified ${\mathrm{tRNA}}^{\mathrm{Arg}}$ corresponding to labels for states marked in the free energy profile (**A**). In each snapshot, folded domains are uniquely colored while the unfolded domains are shown in a light gray color, and G27^*^ in modified ${\mathrm{tRNA}}^{\mathrm{Arg}}$ is highlighted in a space-filling representation (orange). The fractions for states $I_{2}$ and $I_{2}^{\prime }$ (as measured in %) denote the percentage of trajectories in which a specific conformation was observed. (**C**) Distributions of the fraction of native contacts in the *tsl* domain ($Q_{tsl}$) vs. the fraction of native contacts in the *dsl* domain ($Q_{dsl}$) computed from structure-based simulations for the modified ${\mathrm{tRNA}}^{\mathrm{Arg}}$ with labels for the intermediate states ($I_{2}$, $I_{2}^{\prime }$, and $I_{3}$) corresponding to configurations in (**A**,**B**).

**Figure A1:**
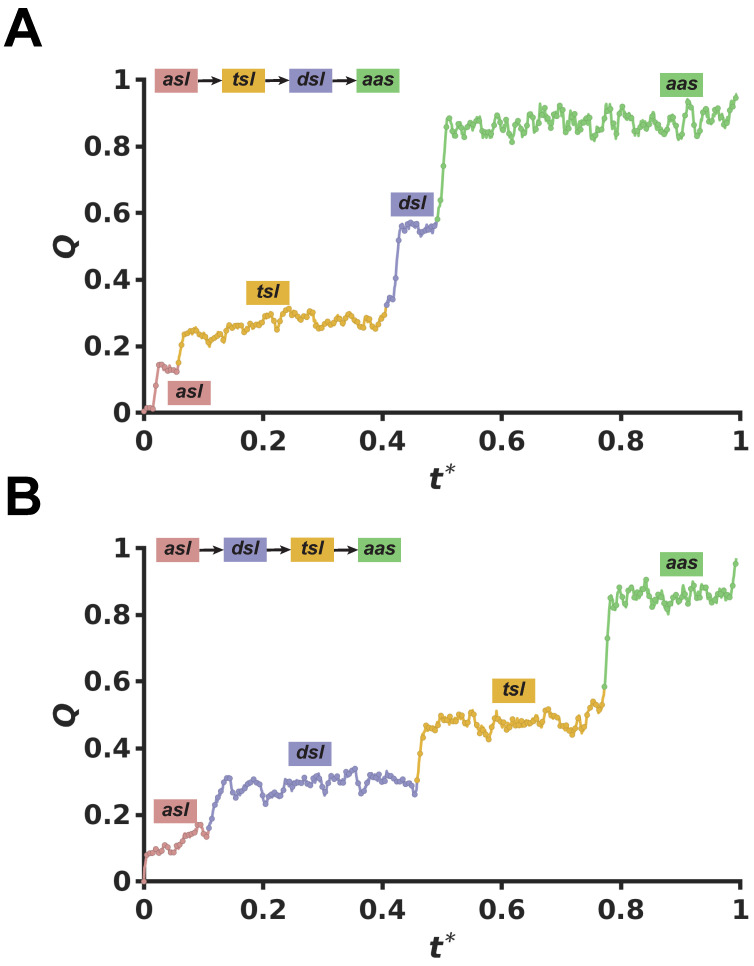
**Representative folding trajectories of modified ${\mathrm{tRNA}}^{\mathrm{Arg}}$**.*Q* is plotted as a function of reduced time ($t^{*}=t/t_{f}$ where $t_{f}$ is the total simulation time) and colored according to the structural domain that folds, illustrating processive folding of (**A**) *asl*→*tsl*→*dsl*→*aas* domains and (**B**) of *asl*→*dsl*→*tsl*→ *aas* domains. See Figure 2 for representative snapshots highlighting these transitions.

Based on conformations sampled during G$\bar{o}$-model simulations, we found that the folding of modified ${\mathrm{tRNA}}^{\mathrm{Arg}}$ was hierarchical, processive and stepwise with each individual domain folding in a particular order, which can be monitored using the fraction (*Q*) of native contacts (Appendix A Figure A1) and the associated free energy profile as a function of *Q*, F(Q) (Figure 2A). The first step in folding of the modified ${\mathrm{tRNA}}^{\mathrm{Arg}}$ was the formation of the *asl* domain (Figure 2B). During this step, the unfolded ${\mathrm{tRNA}}^{\mathrm{Arg}}$ overcame a free energy barrier (${‡}_{1}$; Figure 2A,B), involving stretching of the initially proximal 3′ and 5′ strands. The outward motions of these strands were coupled with the twisting of the *asl* domain into the folded configuration (${‡}_{1}$ and $I_{1}$; Figure 2B). As a result of this step, ${\mathrm{tRNA}}^{\mathrm{Arg}}$ formed a V-shaped configuration with the 5′ and 3′ strands protruding away from the *asl* domain ($I_{1}$; Figure 2B). Additionally, G27^*^ formed stable base-pairing interactions with A45 similar to the interactions formed between $m^{2,2}G26$ and A44, as observed in previous experimental studies [27,50], providing a stable platform for subsequent steps in the folding of ${\mathrm{tRNA}}^{\mathrm{Arg}}$.

In the next step, we observed that the *tsl* or *dsl* domain folded into a stable stem-loop configuration ($I_{2}$ or $I_{2}^{\prime }$; Figure 2A,B). Based on the free energy values, the configurations with the folded *tsl* or *dsl* domain were slightly more favorable than the configurations with the folded *asl* domain (Figure 2A). Notably, the folding of these stem-loops was not concomitant, as indicated by the map of the fraction of native contacts in the *tsl* domain ($Q_{tsl}$) vs. in the *dsl* domain ($Q_{dsl}$) (Figure 2C). If the *tsl* and *dsl* domains folded concomitantly, we would expect to observe a higher density of the concurrently folded configurations along the diagonal of the map (Figure 2C). However, we only observed regions with a higher population of native contacts in the upper left and lower right corners of the map ($I_{2}$ and $I_{2}^{\prime }$; Figure 2C), which indicates a stepwise mechanism of folding of these stem-loops. Thus, the folding of one of the stem-loops (e.g., *tsl*) led to the folding of the other unfolded stem-loop (e.g., *dsl*), and vice versa (${‡}_{2}$; Figure 2B). Furthermore, we observed a nearly equal likelihood of $I_{2}$/$I_{2}^{\prime }$ states in simulations that initially proceeded through the folding of the *tsl* domain ($I_{2}$; 51%) compared to those simulations that proceeded through the folding of the *dsl* domain ($I_{2}^{\prime }$; 49%). This implies that the modified ${\mathrm{tRNA}}^{\mathrm{Arg}}$ can adopt both of these configurations with nearly equal probability.

As a result of the *tsl* and *dsl* folding, a four-way junction structure was formed with the three folded stem loops (*asl*, *dsl*, *tsl*) and the still unfolded *aas* domain ($I_{3}$; Figure 2B). Subsequently, ${\mathrm{tRNA}}^{\mathrm{Arg}}$ transitioned to the native (folded) state, which is the most favorable configuration, as indicated by the free energy profile of the modified ${\mathrm{tRNA}}^{\mathrm{Arg}}$ (Figure 2A). The four-way junction state is separated from the folded state by a free energy barrier (Figure 2A) since the transition is coupled with further conformational rearrangements within the four-way junction structure. Specifically, the *dsl* domain stacked on the *asl* domain, while simultaneously the nucleotides of the *dsl* domain formed base-pairing interactions with the nucleotides of the *vl* domain (Figure 2B). These rearrangements resulted in the formation of the “elbow-like” platform for further stacking of the *tsl* domain. The stacking of the *tsl* domain over the *dsl* domain corresponded to a transition mediated by the free energy barrier (${‡}_{3}$; Figure 2A,B). After that, the *aas* nucleotides formed base-pairing interactions with each other, resulting in the folded (native) state of ${\mathrm{tRNA}}^{\mathrm{Arg}}$ ($I_{3}$; Figure 2A,B). Overall, the folding of the modified ${\mathrm{tRNA}}^{\mathrm{Arg}}$ proceeded along two equally probable pathways that differed only by the order of folding of *tsl* and *dsl* domains (Appendix A Figure A1).

### 2.2. Folding of Unmodified ${\mathrm{tRNA}}^{Arg}$

**Figure 3:**
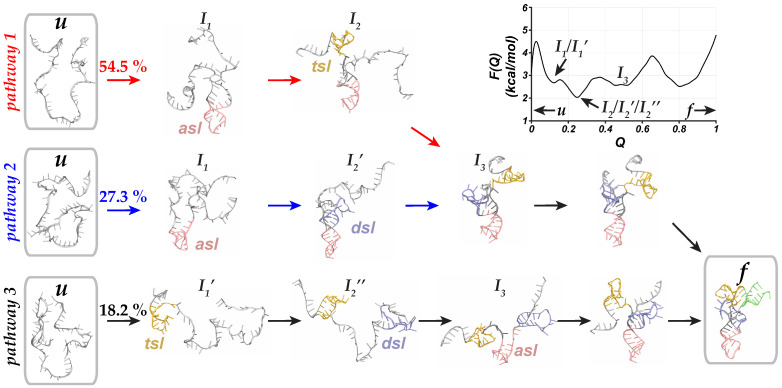
**Folding pathways and thermodynamics of unmodified ${\mathrm{tRNA}}^{\mathrm{Arg}}$**. The structures along three different folding pathways are shown for the unmodified ${\mathrm{tRNA}}^{\mathrm{Arg}}$. In each snapshot, folded domains are uniquely colored and labeled while the unfolded domains are shown in a light gray color. The free energy profile as a function of *Q* is also shown.

**Figure A2:**
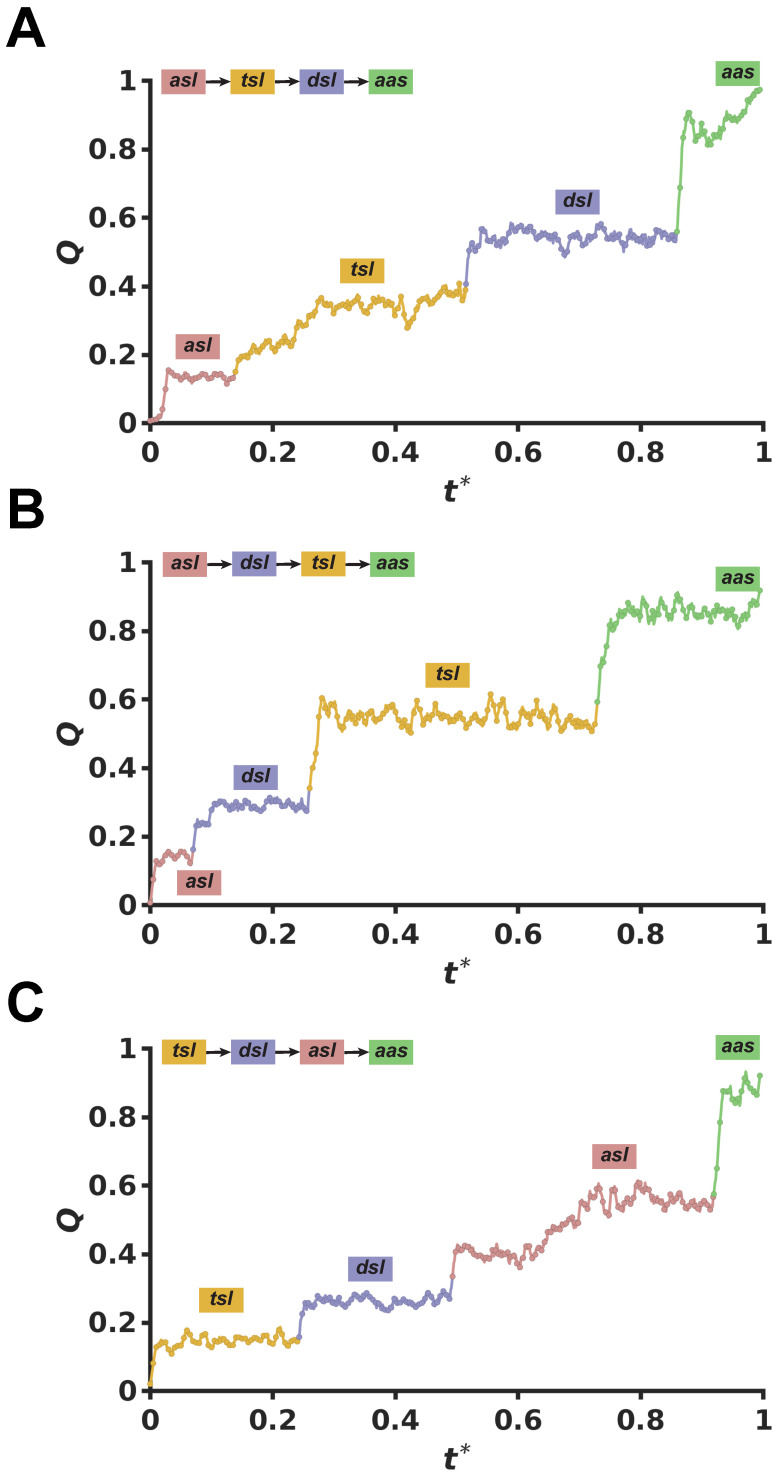
**Representative folding trajectories of unmodified ${\mathrm{tRNA}}^{\mathrm{Arg}}$**. Data similar to Appendix A Figure A1 are shown, illustrating processive folding of (**A**) *asl*→*tsl*→*dsl*→*aas* domains, (**B**) of *asl*→*dsl*→*tsl*→*aas* domains, and (**C**) of *tsl*→*dsl*→*asl*→ *aas* domains. See Figure 3 for representative snapshots highlighting these transitions.

In the absence of methylation at G27, we observed a cooperative stepwise folding mechanism of ${\mathrm{tRNA}}^{\mathrm{Arg}}$ with the formation of the *asl* domain first, followed by the *tsl* (pathway 1) or *dsl* (pathway 2) folding which then stacked onto each other to reach the native state (Figure 3 and Appendix A Figure A2A,B). However, we also observed a new pathway with a distinct folding order from the folding mechanism of the modified ${\mathrm{tRNA}}^{\mathrm{Arg}}$ (pathway 3; Figure 3 and Appendix A Figure A2C). Specifically, in this pathway we first observed the folding of the *tsl* domain, diverging from the previously established folding order in which the *asl* domain always folded first ($I_{1}$ and $I_{1}^{\prime }$; Figure 3). Furthermore, the folding of the *tsl* domain was followed by the folding of the *dsl* domain, while the *asl* domain, which links the *dsl* and *tsl* domains, remained elongated and unfolded ($I_{2}^{\prime \prime }$; Figure 3). This was distinct from pathways 1 and 2 in which the *asl* domain adopted a folded configuration with subsequent folding of the *tsl* or *dsl* domains ($I_{2}$ and $I_{2}^{\prime }$; Figure 3). Notably, these intermediate partially folded configurations of unmodified ${\mathrm{tRNA}}^{\mathrm{Arg}}$, from each of the three possible folding pathways, adopted thermodynamically favorable configurations in the free energy profile ($I_{2}$, $I_{2}^{\prime }$, and $I_{2}^{\prime \prime }$; Figure 3).

Overall, although the fraction of trajectories along the folding pathway 1 (*asl* →*tsl*→*dsl*) was similar to the corresponding pathway for modified ${\mathrm{tRNA}}^{\mathrm{Arg}}$, pathway 2 (*asl*→*dsl*→*tsl*) was less frequently observed in the folding ensemble, with only 27.3% of G$\bar{o}$-model simulations proceeding along pathway 2 (Figure 3). These folding simulations were routed through pathway 3 (*tsl*→*dsl*→*asl*) which was observed in 18.2% of simulations (Figure 3). Thus, while the folding of the unmodified ${\mathrm{tRNA}}^{\mathrm{Arg}}$ was also cooperative and hierarchical (Appendix A Figure A2), it explored a more diverse ensemble of folding pathways and involved stable and partially-folded intermediate configurations. Furthermore, the folded state of the unmodified ${\mathrm{tRNA}}^{\mathrm{Arg}}$ was thermodynamically less favored than the partially-folded intermediate configurations (Figure 3).

### 2.3. Local Dynamics Around G27^*^ During ${\mathrm{tRNA}}^{Arg}$ Folding

**Figure A3:**
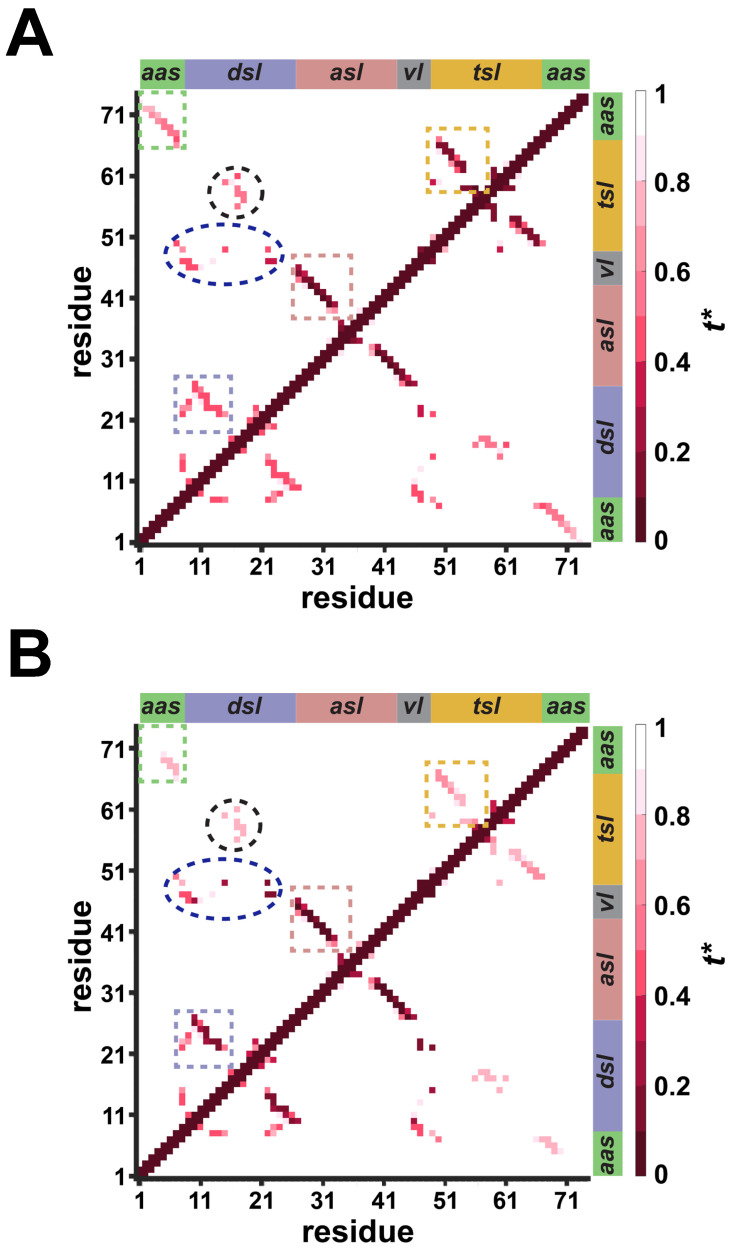
**Residue–residue contact maps from representative folding trajectories of modified ${\mathrm{tRNA}}^{\mathrm{Arg}}$**. Residue–residue interactions are colored according to the reduced time $t^{*}$ (see Appendix A Figure A1), illustrating the temporal order of interaction formation during folding of (**A**) *asl*→*tsl*→*dsl*→*aas* domains and (**B**) of *asl*→*dsl*→*tsl*→ *aas* domains. The white color signifies no interactions between residues were observed throughout the simulation. Structural domains are indicated along both axes. Highlighted regions denote intra-domain interactions (boxed regions) and inter-domain interactions (circled regions).

We further analyzed the residue–residue interactions to determine additional structural details regarding the folding pathways for the modified ${\mathrm{tRNA}}^{\mathrm{Arg}}$. In both representative folding pathways, interactions were first formed in the *asl* domain (Appendix A Figure A3), followed by a progressive formation of residue–residue contacts in the remaining structural domains, consistent with the increase in the *Q* (Appendix A Figure A1). In addition to observing the formation of the intra-domain contacts, we also observed inter-domain interactions between the *dsl* and the *tsl*/*vl* domains (circled regions; Appendix A Figure A3) which signifies tertiary organization of the modified ${\mathrm{tRNA}}^{\mathrm{Arg}}$. Overall, these interaction maps further highlight that modified ${\mathrm{tRNA}}^{\mathrm{Arg}}$ folds through two hierarchical pathways in which the formation of the individual stem loops precedes the formation of long-range tertiary contacts.

**Figure A4:**
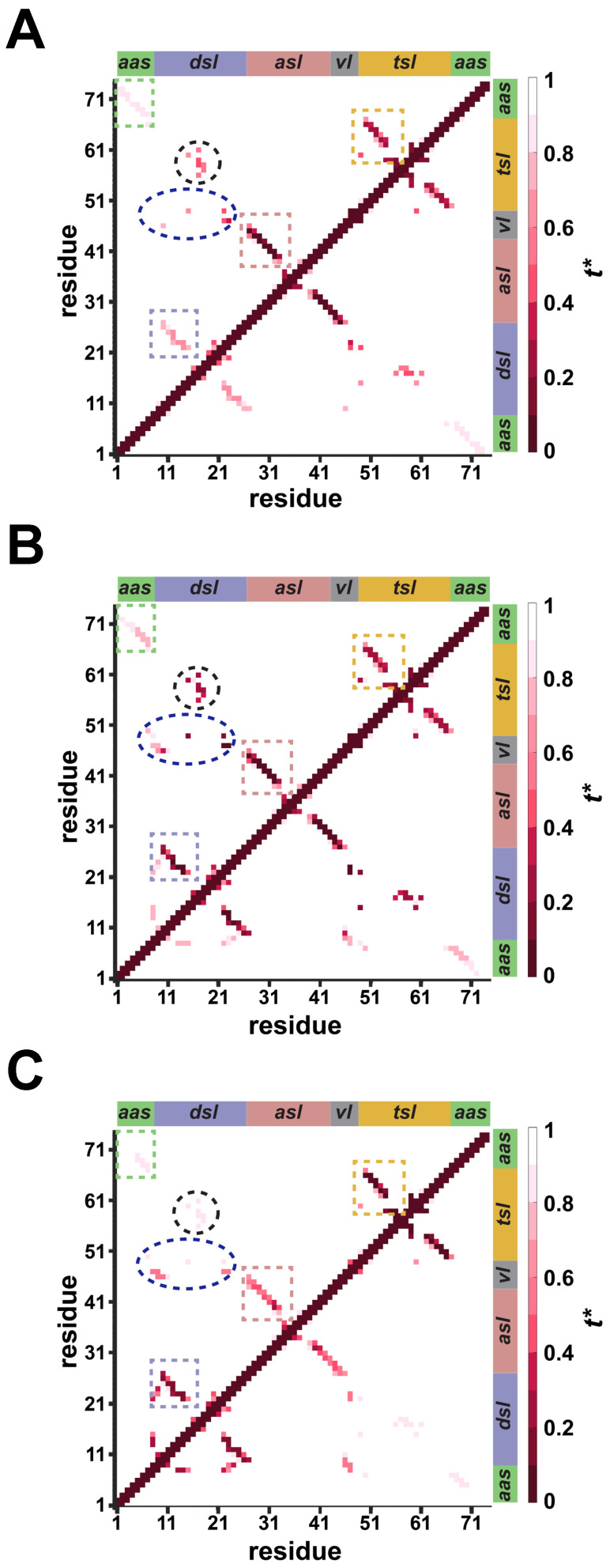
**Residue–residue contact maps from representative folding trajectories of unmodified ${\mathrm{tRNA}}^{\mathrm{Arg}}$**. Data similar to Appendix A Figure A3 are shown, illustrating the formation of residue–residue interactions via the folding of (**A**) *asl*→*tsl*→*dsl*→*aas* domains, (**B**) of *asl*→*dsl*→*tsl*→*aas* domains, and (**C**) of *tsl*→*dsl*→*asl*→ *aas* domains.

The residue–residue interaction maps of the unmodified ${\mathrm{tRNA}}^{\mathrm{Arg}}$ demonstrated the sequential folding of the structural domains via three distinct pathways (Appendix A Figure A4) consistent with increase in the *Q* (Appendix A Figure A2). Compared with the modified ${\mathrm{tRNA}}^{\mathrm{Arg}}$, some of the inter-domain interactions, specifically between the *dsl* and the *tsl*/*vl* junction region (blue circles in Appendix A Figure A4), are generally less distinct and defined, indicating a potentially less cooperative long-range assembly. These interaction maps further confirm the presence of three folding pathways in the unmodified ${\mathrm{tRNA}}^{\mathrm{Arg}}$.

**Figure 4:**
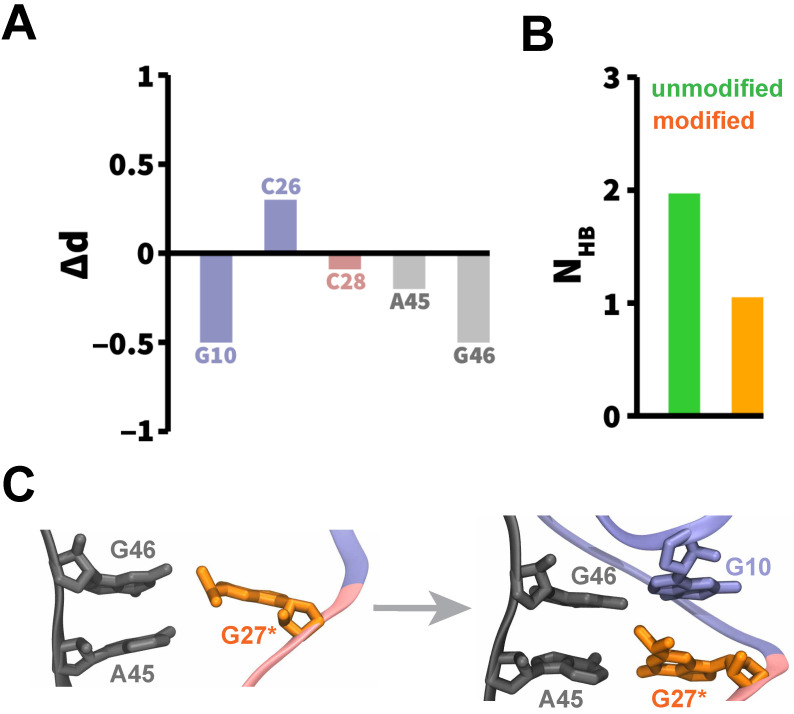
Structural interactions of G27^*^ during the folding of ${\mathrm{tRNA}}^{\mathrm{Arg}}$. (**A**) Differences in center-of-mass to center-of-mass distances ($\Delta d=d_{\mathrm{modified}}-d_{\mathrm{unmodified}}$) between the G27 base and the neighboring bases, computed across all simulations after folding of the relevant structural domains. Bars are colored based on the domain color (see Figure 1) where each nucleotide is located. (**B**) Average number of hydrogen bonds ($N_{\mathrm{HB}}$) formed between the G27 and A45 bases in the unmodified and modified ${\mathrm{tRNA}}^{\mathrm{Arg}}$, computed across all simulations after the formation of the G27-A45 base pair. (**C**) Representative snapshots highlighting local interactions formed by the G27^*^ nucleotide (orange) with neighboring residues during folding of the modified ${\mathrm{tRNA}}^{\mathrm{Arg}}$.

**Figure A5:**
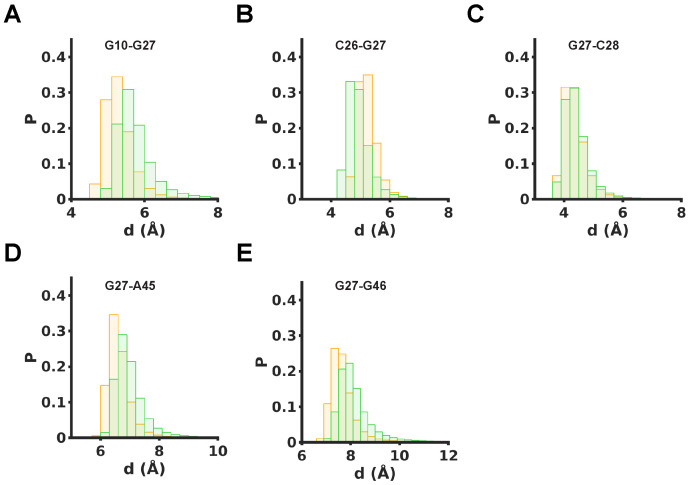
**Distributions of residue–residue distances around the G27 nucleotide**. Probability distributions of center-of-mass to center-of-mass distances (d) between G27 and neighboring residues computed across all simulations after folding of the relevant structural domains for the unmodified (green) and modified (orange) ${\mathrm{tRNA}}^{\mathrm{Arg}}$. The shown residue pairs are (**A**) G10-G27, (**B**) C26-G27, (**C**) G27-C28, (**D**) G27-A45, and (**E**) G27-G46.

We further conducted the local structural analysis of G27 in the unmodified and modified ${\mathrm{tRNA}}^{\mathrm{Arg}}$ to identify the effects of the modification on the local dynamics of tRNA during folding. The center-of-mass differences shown in Figure 4A indicate that methylation at G27 induces measurable rearrangements in the immediate nucleotide environment after folding of the relevant structural domains. Specifically, negative Δd values for G10, A45, and G46 indicate that these residues are closer to the modified G27 nucleotide, relative to the unmodified variant, whereas a positive Δd value for C26 suggests that this nucleotide is slightly displaced away from G27^*^ relative to the unmodified G27. The full distance distributions for these residue pairs are shown in Appendix A Figure A5, which show systematic shifts in the distributions, thereby indicating that the observed differences reflect persistent modification-dependent local structural reorganization.

Additionally, we computed the average number of hydrogen bonds formed between G27 and A45 due to base pairing (Figure 4B). We observed that the average number of hydrogen bonds formed between residues G27 and A45 is reduced in the modified ${\mathrm{tRNA}}^{\mathrm{Arg}}$ relative to the unmodified variant, suggesting that methylation perturbs direct base-pairing interactions while enabling alternative local packing arrangements. Representative snapshots from the modified ${\mathrm{tRNA}}^{\mathrm{Arg}}$ simulations (Figure 4C) showed that G27^*^ first interacted with the nucleotides from the *vl* domain (A45 and G46 nucleotides), followed by the formation of stacking interactions with the G10 nucleotide from the *dsl* domain. As a result, the nucleotides G10, G27, A45, and G46 maintained a stable network of interactions with each other (Figure 4C). Together, these results suggest that the modification reorganizes the local interaction network around residue G27^*^ during the folding of ${\mathrm{tRNA}}^{\mathrm{Arg}}$.

## 3. Discussion

Our findings on tRNA folding are consistent with the general notion that RNA molecules have rugged folding landscapes [51]. Therefore, the folding of large RNA molecules such as tRNA is considered hierarchical and processive, where the assembly of secondary structure elements precedes the formation of tertiary interactions [51,52]. The formation of secondary structure elements is initially slowed due to the charged nature of the RNA backbone, potentially due to repulsion between phosphate groups, but once the first base-pairing interactions are formed, the folding of other elements proceeds rapidly and cooperatively [1,2]. Furthermore, it is known that even a small change in RNA sequence or structure through mutation or modification could lead to altered folding pathways [51,52]. In this work, we also observed the folding of ${\mathrm{tRNA}}^{\mathrm{Arg}}$ with/without modification to proceed through a hierarchical mechanism that follows distinct pathways, consistent with the current understanding of tRNA folding [2].

We observed that the modified ${\mathrm{tRNA}}^{\mathrm{Arg}}$ system followed a narrower set of folding pathways. The folding of the first domain (*asl*) was separated from the unfolded configuration by a free energy barrier (Figure 2A). To overcome this barrier, ${\mathrm{tRNA}}^{\mathrm{Arg}}$ transitioned through various unfolded configurations until the first base-pairing interactions formed in the *asl* domain (Figure 2B). This local folding of the *asl* domain in the modified ${\mathrm{tRNA}}^{\mathrm{Arg}}$ simultaneously led to the folding of the other two stem loops (*tsl* and *dsl*; Figure 2A,B). This is consistent with experimental findings that the folding of the first secondary structure element (*asl*) simultaneously causes the rapid and cooperative formation of other stem loops [2]. After that, the individually folded stem loops were stacked on top of each other, adopting a thermodynamically favored folded state of ${\mathrm{tRNA}}^{\mathrm{Arg}}$ (Figure 2A). Notably, the intermediate partially folded configurations of the modified ${\mathrm{tRNA}}^{\mathrm{Arg}}$ corresponded to local free energy minima ($I_{1}$, $I_{2}$, $I_{2}^{\prime }$, and $I_{3}$; Figure 2A) which were thermodynamically less favored than fully folded ${\mathrm{tRNA}}^{\mathrm{Arg}}$. This indicated a thermodynamic preference of the modified ${\mathrm{tRNA}}^{\mathrm{Arg}}$ toward a fully folded configuration.

However, without modification, the intermediate partially-folded configurations were more thermodynamically stable than the unfolded or folded states of the unmodified ${\mathrm{tRNA}}^{\mathrm{Arg}}$ (Figure 3). Specifically, the unmodified ${\mathrm{tRNA}}^{\mathrm{Arg}}$ adopted the most thermodynamically favored partially-folded configuration with two folded stem loops ($I_{2}$, $I_{2}^{\prime }$, and $I_{2}^{\prime \prime }$; Figure 3). Furthermore, other partially-folded ${\mathrm{tRNA}}^{\mathrm{Arg}}$ configurations ($I_{1}$, $I_{1}^{\prime }$; Figure 3) were also thermodynamically more favored than the unfolded/folded states of the unmodified ${\mathrm{tRNA}}^{\mathrm{Arg}}$. As a result, unmodified ${\mathrm{tRNA}}^{\mathrm{Arg}}$ populated partially folded intermediate states more frequently, potentially delaying the transition to the four-way junction structure with three folded stem loops (*asl*, *tsl*, and *dsl*; Figure 3), contrary to what was observed in the folding of modified ${\mathrm{tRNA}}^{\mathrm{Arg}}$ (Figure 2).

Furthermore, we identified a distinct folding pathway for unmodified ${\mathrm{tRNA}}^{\mathrm{Arg}}$ system in which the folding was initiated by the formation of the *tsl* domain rather than the *asl* domain ($I_{1}^{\prime }$; Figure 3). As a result, an elongated rod-shaped configuration was formed which was not observed in modified ${\mathrm{tRNA}}^{\mathrm{Arg}}$ simulations ($I_{1}^{\prime }$; Figure 3). This configuration could further disrupt the folding of the *asl* domain, leading to alternate pathways with partially-folded/intermediate configurations that can broaden the ensemble of folding pathways. Thus, the greater number of folding pathways observed for the unmodified ${\mathrm{tRNA}}^{\mathrm{Arg}}$ indicates an increase in the structural diversity of intermediate configurations, which could enhance the likelihood of alternate partially folded states.

Overall, we showed that double-methylation of G27 facilitates the folding process of ${\mathrm{tRNA}}^{\mathrm{Arg}}$ (and possibly other tRNA molecules) in a more controlled, thermodynamically favored, and hierarchical manner to potentially avoid alternate tRNA configurations. In summary, our results provide insights into the effect of methylation on the folding of ${\mathrm{tRNA}}^{\mathrm{Arg}}$ and have potential broader implications for the folding of other nuclear-encoded tRNA molecules. We also anticipate that the configurations from our structure-based simulations could be useful for seeding future simulation studies of tRNA folding.

## 4. Materials and Methods

### 4.1. Descriptions of the Unfolded and Folded States

**Figure A6:**
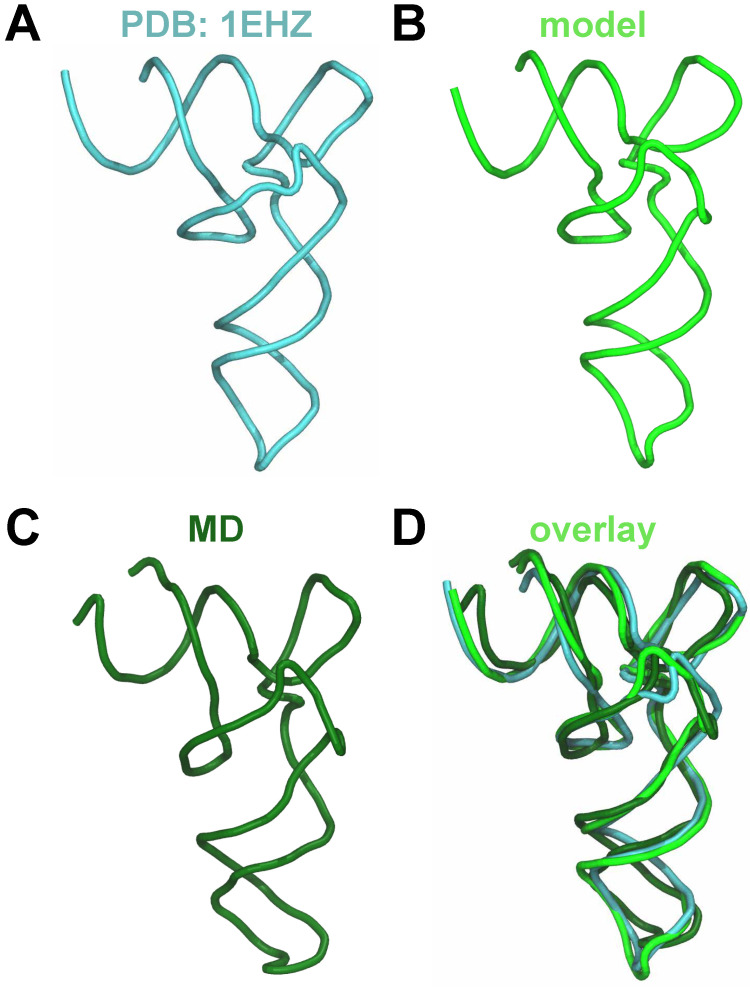
**Structural comparison**. (**A**) Conserved L-shaped tRNA fold is shown based on a crystal structure of tRNA (PDB ID: 1EHZ) [56], (**B**) the ${\mathrm{tRNA}}^{\mathrm{Arg}}$ structure modeled using trRosettaRNA for this study, (**C**) the average structure obtained from a 1 μs-long all-atom MD simulation of the solvated and ionized model shown in (**B**), and (**D**) an overlay of structures shown in (**A**–**C**).

An unmodified structural model of ${\mathrm{tRNA}}^{\mathrm{Arg}}$ was generated using the sequence of ${\mathrm{tRNA}}^{\mathrm{Arg}}$ reported in the GtRNAdb repository [53,54] and the RNA structure prediction software trRosettaRNA v1.0 [55]. We also compared this model with the canonical tRNA structure [56] which showed similar folds for all structural domains (Appendix A Figure A6). Furthermore, we obtained an average structure from a 1 μs-long all-atom MD simulation with CHARMM36 force field [57,58,59] of the solvated and ionized structural model, and compared with predicted model and crystal structure (Appendix A Figure A6). We observed the average structure to remain in close agreement with the predicted model and the canonical tRNA fold (Appendix A Figure A6D). Collectively, these comparisons support the use of the predicted structure for further simulations. The Molefacture plugin in the Visual Molecular Dynamics (VMD v1.9.3) software [60] was used to introduce $m^{2,2}G$ modification in the G27 nucleotide of the modeled ${\mathrm{tRNA}}^{\mathrm{Arg}}$ structure, thus generating two models of ${\mathrm{tRNA}}^{\mathrm{Arg}}$, one each in the unmodified and modified states. The folded structures for each ${\mathrm{tRNA}}^{\mathrm{Arg}}$ model (unmodified and modified) were subsequently used as the native (final) states in G$\bar{o}$-model simulations.

Unfolded configurations were generated by performing all-atom MD simulations in explicit solvent at temperatures (between ∼550 K and ∼700 K) above the known range of the melting temperature of some tRNA species, typically between 323 K and 353 K [61,62,63,64]; the exact melting temperature of the studied tRNA is currently unknown. These unfolded configurations with no native contacts were used as initial structures for G$\bar{o}$-model simulations. In total, 120 unique unfolded configurations were generated for G$\bar{o}$-model simulations, 60 for each of the two ${\mathrm{tRNA}}^{\mathrm{Arg}}$ systems (modified and unmodified). Thus, the trRosettaRNA software was used only to obtain the native (folded) reference state with defined native contacts, whereas the high-temperature MD simulations were used only to generate unfolded initial conformations with no native contacts for G$\bar{o}$-model simulations.

### 4.2. All-Atom G$\bar{o}$-Model Simulations

We used all-atom G$\bar{o}$-model simulations [40,41,42,43] to characterize the folding mechanism of ${\mathrm{tRNA}}^{\mathrm{Arg}}$ with and without the $m^{2,2}G$ modification. By construction, only native contacts (present in the native state) are considered attractive while all non-native contacts (not present in the native state) are mutually repulsive, thereby making the potential energy function suitable for studying the folding toward the native structure [39]. Thus, physics-based non-bonded interactions are replaced by a potential energy function which has been employed in prior RNA folding studies [48,49] based on the native structure:

(1) $$V_{G\bar{o}}=4\epsilon_{i,j}^{native}\left[{\left(\frac{\sigma_{i,j}}{r_{i,j}}\right)}^{12}-{\left(\frac{\sigma_{i,j}}{r_{i,j}}\right)}^{10}\right]+4\epsilon_{i,j}^{non-native}{\left(\frac{\sigma_{i,j}}{r_{i,j}}\right)}^{12}$$

Here, $\epsilon_{i,j}^{native}$ and $\epsilon_{i,j}^{non-native}$ denote the potential well depths corresponding to native and non-native contacts, respectively, $r_{i,j}$ represents the pairwise distance between any two atoms *i* and *j*, and $\sigma_{i,j}$ represents the pairwise distance between the atoms *i* and *j* in the native (folded) state. The $\epsilon_{i,j}^{native}$ and $\epsilon_{i,j}^{non-native}$ were set to 0.14 and 0.01, respectively, as employed in prior simulation studies of RNA folding [48,49]. Based on Equation (1), the attractive interactions are only assigned to contact pairs found in the native state within a specific distance cutoff, whereas all non-native contacts are mutually repulsive. In our study, native contacts are defined as any pairs of heavy atoms within a distance of 4 Å in the native (folded) state, as also done in previous simulation studies of RNA systems [48,49]. Per the simulation protocol, we conducted all Gō-model simulations in the absence of solvent, while the ionic effects are implicitly incorporated through the choice of $\epsilon_{i,j}^{native}$ and $\epsilon_{i,j}^{non-native}$ [48]. All simulations were conducted using the Nanoscale Molecular Dynamics (NAMD v 2.14) software package [65]. All bonded interactions (bonds, angles, and dihedral angles) are adopted from the CHARMM36 force-field, which has updated parameter sets for the modified nucleotides [57,58,59].

Multiple independent G$\bar{o}$-model simulations were initiated from a different unfolded configuration chosen from an ensemble of unfolded ${\mathrm{tRNA}}^{\mathrm{Arg}}$ conformations. Due to their central role in G$\bar{o}$-model simulations, the native contacts of each structural domain, as well as of the full ${\mathrm{tRNA}}^{\mathrm{Arg}}$, were used to monitor the progression of each simulation toward the native state, and to further categorize the corresponding type of folding pathway based on the observed sequence of domain formation. Specifically, these simulations were conducted until the folding of ${\mathrm{tRNA}}^{\mathrm{Arg}}$ was achieved, corresponding to the formation of over 80% (*Q* > 0.8) of all native contacts, as also used in other folding studies [46,49]. All simulations were conducted at a temperature of 353 K as the upper value of the known tRNA melting temperature range to enhance the sampling of the folding landscape. Simulating near or above the folding transition temperature promotes frequent transitions between folded and unfolded states, enabling efficient characterization of folding pathways and intermediates [66]. The unfolded configurations were characterized by *Q* < 0.2, while the intermediate configurations were characterized by 0.2 < *Q* < 0.8. In total, we generated 120 G$\bar{o}$-model simulations, 60 for each of the two ${\mathrm{tRNA}}^{\mathrm{Arg}}$ system (modified and unmodified). The free energy (*F*) was computed as a function of *Q*: $F\left(Q\right)=-k_{B}TlnP\left(Q\right)$. It is important to note that the G$\bar{o}$-model potential is inherently biased toward the predefined native structure [39,48]. Furthermore, the G$\bar{o}$-model potential does not explicitly represent the full-range of non-native interactions which may form during the course of a simulation. However, despite these limitations, the G$\bar{o}$-model framework remains useful for characterizing dominant folding pathways, intermediate states, and transition barriers in biomolecular systems [44,45,46,47,48,49].

## 5. Conclusions

We used structure-based G$\bar{o}$-model simulations to characterize the folding mechanism of a ${\mathrm{tRNA}}^{\mathrm{Arg}}$ isodecoder, with and without a chemical modification at the G27 nucleotide. Our results suggest that the folding of the modified ${\mathrm{tRNA}}^{\mathrm{Arg}}$ proceeds in a cooperative manner with a specific order of folding for secondary structure elements (*asl*→*tsl*/*dsl*→*aas*), resulting in a thermodynamically favored folded structure. In the absence of modification at G27, we observed a broader ensemble of folding pathways, characterized by thermodynamically favored partially folded configurations, which likely perturb the folding process and facilitate the formation of alternate folding pathways. Thus, the chemical modification at G27 enables the folding of tRNA isodecoder along a more hierarchical folding pathway. The folding mechanism of ${\mathrm{tRNA}}^{\mathrm{Arg}}$ is consistent with the current understanding of the hierarchical folding mechanism in other RNA molecules. Overall, our results provide insights into the effect of methylation on ${\mathrm{tRNA}}^{\mathrm{Arg}}$ folding and have potential broader implications for the folding of other nuclear-encoded tRNA molecules. Furthermore, the broader principles identified in this work may also be relevant to mitochondrial tRNAs, particularly those capable of adopting a canonical tRNA configuration, but further work will be needed to identify system specific features of mitochondrial tRNAs.

## Data Availability

The original contributions presented in this study are included in the article. Further inquiries can be directed to the corresponding author.

## Abbreviations

The following abbreviations are used in this manuscript:

*aas*	Amino Acid Acceptor Stem
*asl*	Anticodon Stem Loop
CHARMM	Chemistry at Harvard Macromolecular Mechanics
*dsl*	D-stem Loop
mRNA	Messenger Ribonucleic Acid
MD	Molecular Dynamics
NAMD	Nanoscale Molecular Dynamics
tRNA	Transfer Ribonucleic Acid
${\mathrm{tRNA}}^{\mathrm{Arg}}$	Arginine Transfer Ribonucleic Acid
*tsl*	T-stem Loop
*vl*	Variable Loop
